# Computational Fluid Dynamics Study of Bifurcation Aneurysms Treated with Pipeline Embolization Device: Side Branch Diameter Study

**DOI:** 10.1007/s40846-015-0046-3

**Published:** 2015-06-30

**Authors:** Abraham Yik-Sau Tang, Wai-Choi Chung, Eric Tian-Yang Liu, Jie-Qiong Qu, Anderson Chun-On Tsang, Gilberto Ka-Kit Leung, Kar-Ming Leung, Alfred Cheuk-Hang Yu, Kwok-Wing Chow

**Affiliations:** Department of Mechanical Engineering, The University of Hong Kong, Pokfulam, Hong Kong, 999077 China; Division of Neurosurgery, Department of Surgery, Li Ka Shing Faculty of Medicine, The University of Hong Kong, Queen Mary Hospital, Pokfulam, Hong Kong, 999077 China; Department of Neurosurgery, Kwong Wah Hospital, Waterloo Road, Hong Kong, 999077 China; Medical Engineering Program, Department of Electrical and Electronic Engineering, The University of Hong Kong, Pokfulam, Hong Kong, 999077 China

**Keywords:** Intracranial aneurysm, Endovascular treatment, Computational fluid dynamics (CFD)

## Abstract

An intracranial aneurysm, abnormal swelling of the cerebral artery, may lead to undesirable rates of mortality and morbidity upon rupture. Endovascular treatment involves the deployment of a flow-diverting stent that covers the aneurysm orifice, thereby reducing the blood flow into the aneurysm and mitigating the risk of rupture. In this study, computational fluid dynamics analysis is performed on a bifurcation model to investigate the change in hemodynamics with various side branch diameters. The condition after the deployment of a pipeline embolization device is also simulated. Hemodynamic factors such as flow velocity, pressure, and wall shear stress are studied. Aneurysms with a larger side branch vessel might have greater risk after treatment in terms of hemodynamics. Although a stent could lead to flow reduction entering the aneurysm, it would drastically alter the flow rate inside the side branch vessel. This may result in side-branch hypoperfusion subsequent to stenting. In addition, two patient-specific bifurcation aneurysms are tested, and the results show good agreement with the idealized models. Furthermore, the peripheral resistance of downstream vessels is investigated by varying the outlet pressure conditions. This quantitative analysis can assist in treatment planning and therapeutic decision-making.

## Introduction

An intracranial aneurysm is a severe cerebrovascular disorder where a portion of the artery wall becomes weakened and dilated. It commonly occurs near arterial bifurcations in the Circle of Willis [1, 2]. The weakened part may rupture, resulting in intracranial hemorrhage. The mortality rate of subarachnoid hemorrhage due to aneurysmal rupture can reach 50 %. The occurrence rate of intracranial aneurysm is about 2–5 % in the population, and thus deserves careful investigation [3].

A popular endovascular treatment for intracranial aneurysms is coil embolization [4]. By filling the aneurysm lumen with metallic coils, flow stasis can be achieved, promoting thrombus formation. Catheter-delivered, self-expandable, flow-diverting stents have become widely used for endovascular treatment for cerebral aneurysms. The underlying principle of this stent deployment mechanism is to divert blood flow from the aneurysm by covering the orifice. This action can disrupt the pulsatile blood flow within an aneurysm sac to the point of stagnation and obliteration, promoting the formation of thrombus. Flow-diverting stents have a superior aneurysm occlusion rate compared to that of coil embolization, with comparable complication rates. Coil embolization in aneurysms carries a long-term risk of incomplete occlusion and delayed recurrence, especially at the neck of the aneurysm. It is also unsuitable for aneurysms with complex morphologies, such as wide-necked or fusiform aneurysms. Flow-diverting stents can overcome these limitations and are thus suitable for a wide range of aneurysms. The occlusion rate is satisfactory, with up to an 80–95 % success rate in 1 year [5]. A recent systematic review of pipeline stent (a type of flow-diverting stent) treatment of intracranial aneurysms showed that the 6-month aneurysm obliteration rate was 82.9 % [6].

Since aneurysms are often located near a bifurcation, the ostium of the adjacent side branch will be covered by the deployed flow-diverting stent and blood flow into such a branch may be affected, possibly leading to ischemic complications. This alteration of blood flow inside the aneurysm and nearby vasculature by the stent must be considered carefully, since there have been reports of immediate or delayed occlusion of covered side branches subsequent to such stent deployment, in particular the ophthalmic artery and lenticulostriate arteries arising from A1 and M1 segments of the anterior cerebral artery and the middle cerebral artery, respectively [7, 8]. Flow-diverting stents also facilitate the regrowth of cells over them to achieve remodeling of the arterial wall [9].

There are two types of commercially available flow-diverting stent, namely the pipeline embolization device (PED) (Covidien Vascular Therapies, Mansfield, MA, USA) and the Silk stent (Balt, Montmorency, France) [10]. The present study focuses on the former. In April 2011, the PED was granted Food and Drug Administration (FDA) approval for the treatment of intracranial aneurysms [11]. The stent is a cylindrical construct composed of a mesh of 48 individual cobalt chromium and platinum strands, with a surface coverage of 30–35 % [12]. This implies a porosity (empty space on the stent surface divided by the total stent surface area) range of roughly 65–70 %, but physicians can alter the effective porosity by controlling the packing density and number of stents implanted at a given location [7].

**Fig. 1:**
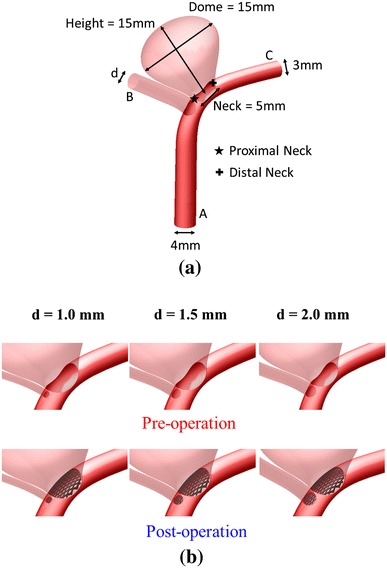
Idealized intracranial aneurysm model considered in this investigation. **a** Geometry and dimensions; *A*, *B*, and *C* respectively denote proximal parent artery, side branch vessel, and distal parent artery. Proximal neck and distal neck region are indicated with *star* and *plus* symbols, respectively. **b** 3D perspective of intracranial aneurysm model with side branch diameter *d* ranging from 1.0 to 2.0 mm. *First row* represents pre-stenting condition, while *second row* describes post-stenting situation

The effect of side branch diameter on hemodynamics was quantitatively studied with idealized and patient-specific bifurcation aneurysm models. The dimension of the side branch vessel was chosen to mimic the posterior communicating artery in real patients. A mean diameter of 1.5 mm has been reported in the literature [13]. Given that the distal parent artery in the present model is 3.0 mm in diameter, three values of side branch vessel diameter, *d*, were tested (*d* = 1.0, 1.5, and 2.0 mm) (Fig. 1).

The issue of post-treatment hemodynamics has been addressed using pipeline stent deployment. Computational fluid dynamics (CFD), a commonly utilized tool in medical engineering [14–16], has been applied to intracranial aneurysms [17]. Several biomechanical factors, including aneurysm geometry and nearby vasculature, have been investigated by considering the pre-operation stage of the aneurysm [18, 19]. Moreover, a high-aspect-ratio aneurysm is generally considered to pose a greater risk [20], and this has been supported by clinical findings [21]. Stent configuration, porosity, and position are all believed to be crucial determining factors of treatment efficacy [4, 22, 23]. In general, a stent with low porosity can reduce the flow entering the aneurysm substantially [23, 24]. However, the flow dynamics associated with changing side branch vessel diameter was not analyzed quantitatively in these earlier works. Studying the hemodynamics with various side branch diameters, especially with stent deployment, can provide useful information in clinical decision-making and treatment planning. Furthermore, utilizing patient-specific bifurcation aneurysm models and comparing them with idealized models can provide further clinical insight.

## Materials and Methods

### Geometrical Models

Idealized intracranial aneurysm models were created using the software SOLIDWORKS 2012 (Dassault Systèmes SolidWorks Corp., Concorde, MA, USA), with the aneurysm being adjacent to an arterial bifurcation. This mimics the anatomy found in many patients [2]. The proximal parent artery (“A” in Fig. 1a) and the distal parent vessel (“C” in Fig. 1a) have diameters of 4 and 3 mm, respectively. The nearby side branch vessel (“B” in Fig. 1a) has a diameter that varies from 1.0 to 2.0 mm [13]. This study focuses on a case with a high aspect ratio (ratio between the aneurysm height and aneurysm neck), which may be associated with a higher risk of rupture [21]. The maximum diameter (assumed to be equal to the height for this model) and the aspect ratio of the aneurysm were set as 15 mm and 3.0, respectively.

After virtual pipeline stenting, the aneurysm neck and the side branch vessel were covered with a stent porosity of roughly 66 % (Fig. 1b). The computational stent had a mesh-like structure, with rhombus-shaped closed cells employed. The stent was postulated to be a rigid device.

**Fig. 2:**
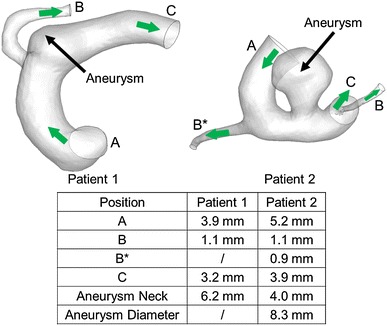
Patient-specific models utilized with aneurysm located near arterial bifurcation. Nearby geometries of two patient-specific models are close to those in idealized models

The results from these idealized models are later compared with patient-specific configurations. Two aneurysms located near the bifurcation of the internal carotid artery (ICA) were selected, where the linear dimensions of the vessels in the nearby vasculature are close to those from the idealized models (Fig. 2). Patient 1 (male; age: 58 years) had a wide-necked aneurysm and Patient 2 (female; age: 75 years) had a saccular aneurysm. Both patients had undergone the pipeline stent treatment. The three-dimensional (3D) computational models were reconstructed from the angiograms using the software Mimics (Materialise, Leuven, Belgium) for CFD analysis. With the computer-aided design software SOLIDWORKS, the virtual stent was deployed in both models. The proximal parent artery and the side branch vessels that originate at the bifurcation and the distal parent vessel are denoted as “A”, “B”, and “C”, respectively, for both patients. For Patient 2, there is another side branch vessel upstream of the aneurysm (denoted as “B*” in Fig. 2).

The computational mesh was generated using the software GAMBIT 2.4.6 (FLUENT, ANSYS, Canonsburg, PA, USA). The size of the spatial grid was selected to be 0.3 mm, and was refined locally to maintain the resolution if necessary. After the deployment of the stent, further refinement of the spatial grids was performed, with the smallest grid size being 0.032 mm. Table 1 lists the number of tetrahedral elements of the models with various side branch diameters and the patient-specific models.

**Table 1:** Number of elements for various side branch diameters and two patient-specific models with pre-stenting (indicated as pre-operation) and post-stenting (indicated as post-operation) conditions

Models	Stent deployment	Side branch diameter *d* (mm)	Number of elements
Idealized	Pre-operation	1.0	1,121,129
		1.5	751,230
		2.0	756,983
	Post-operation	1.0	2,632,483
		1.5	2,746,364
		2.0	2,029,189
Patient 1	Pre-operation	–	539,662
	Post-operation	–	2,265,762
Patient 2	Pre-operation	–	687,787
	Post-operation	–	2,419,143

Mesh independence tests were performed to validate the computational results. As an illustrative example, three sets of data for the *d* = 2.0 mm case with stent deployment were obtained, and the hemodynamic parameters were compared (Table 2). Based on the test results, the model with 2,029,189 grids was taken as sufficiently accurate, since the percentage error between two successive mesh models was within a few percent.

**Table 2:** Mesh independence test results

Stent deployment	Number of elements	Maximum flow velocity at systole (m s^−1^)			Stent migration force at systole (N m^−2^)	Mean volume flow rate (mm^3^ s^−1^)		
		An	SBV	DPA		An	SBV	DPA
Post-operation	1,008,475	0.776	0.646	1.178	43.2	150.8	589.5	2992.1
	2,029,189	0.880	0.675	1.214	47.3	146.2	582.8	3003.6
	2,680,147	0.911	0.680	1.226	48.1	144.3	579.5	3009.2

*d* = 2.0 mm case with stent deployment is taken as illustrative example

*An* aneurysm, *SBV* side branch vessel, *DPA* distal parent artery

### Fluid Mechanics and Boundary Conditions

The numerical simulations were performed using the CFD package FLUENT 6.3.26. The dynamics of the 3D fluid flow is governed by mass conservation (the continuity equation) and momentum consideration (the Navier–Stokes equations). With repeated indices implying summation, the formulations are:

1 $$ \frac{{\partial u_{i} }}{{\partial x_{i} }} = 0 , $$

2 $$ \frac{{\partial u_{i} }}{\partial t} + u_{j} \frac{{\partial u_{i} }}{{\partial x_{j} }} = - \frac{1}{\rho }\frac{\partial p}{{\partial x_{i} }} + \frac{1}{\rho }\frac{{\partial \tau_{ij} }}{{\partial x_{j} }} $$

where *u*_*i*_ (*i* = 1, 2, 3) are the components of the velocity vector, *ρ* is the fluid density, *p* is the pressure, and *τ*_*ij*_ is the normal stress or tangential shear stress. These equations were solved with a time step of 0.001 s. The residual error of continuity was set to 10^−6^. Blood was assumed to be an incompressible Newtonian fluid and treated as a continuum [22], with density and dynamic viscosity values of 1060 kg m^−3^ and 0.0035 kg m^−1^ s^−1^, respectively [25].

**Fig. 3:**
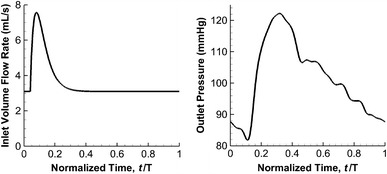
Physiological input for CFD simulations (normalized with respect to cardiac period T): (*left*) pulsatile volume flow rate waveform and (*right*) pressure waveform imposed at inlet and outlets, respectively

A pulsatile volume flow rate profile was imposed at the inlet [26, 27]. A physiologically realistic pressure waveform of 122/82 mmHg (1 mmHg = 133.3 Pa) was prescribed at the outlets (Fig. 3) [27]. The period of a cardiac cycle (T) was taken as 0.8 s (i.e., a heart rate of 75 beats per minute). The third cycle of the computations was used for analysis, as a periodic output had been typically generated then. The properties reported refer to those at the peak systole unless otherwise specified. At the inlet, the maximum Reynolds number attained was 642, and thus the flow was laminar. The no-slip condition was applied at the rigid walls [19]. The Womersley number *α*, which measures the transient effects of the flow [28], is defined as:

3 $$ \alpha = R\sqrt {\frac{\rho \omega }{\mu }} , $$

where *R* is the radius of the vessel, *ω* is the angular frequency, and *μ* is the viscosity (=3.1 here). With these input parameters, the volume influx into this Y-shaped model was 3586.4 mm^3^ s^−1^.

## Results and Discussion

### Study of Side Branch Diameter Using Idealized Models

#### Velocity and Pressure

**Fig. 4:**
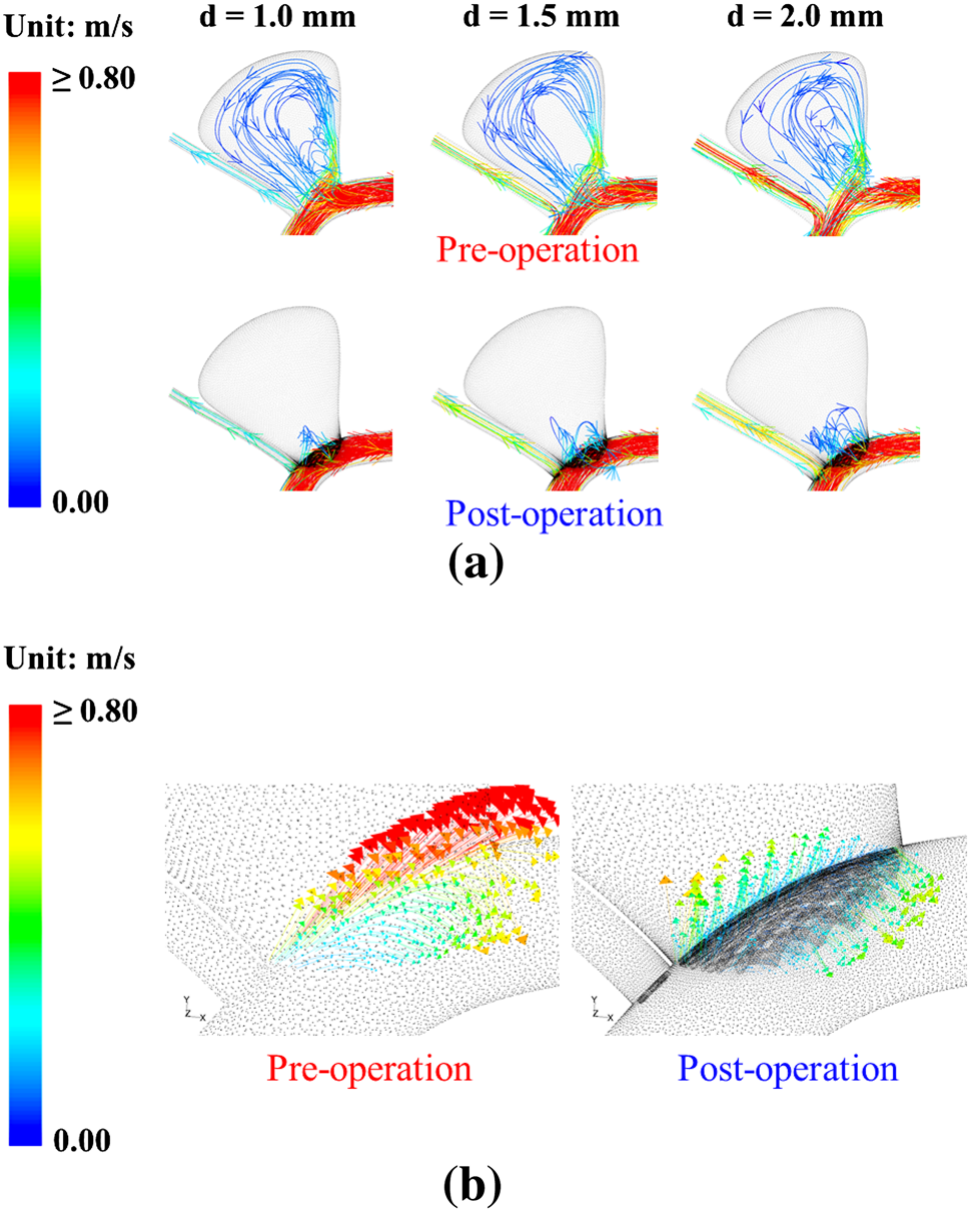
Computational results generated for pre-operation and post-operation models. **a** Streamline plots at peak systole for various side branch diameters are shown. When side branch diameter is small (*d* = 1.0 mm), flow velocity reduction in aneurysm sac after stenting is significant (see also Fig. 5). **b** 3D perspective vector diagrams near aneurysm neck of model with *d* = 1.0 mm

The flow patterns before and after stent placement are shown in Fig. 4a. Before the placement of the stent, flow impingement onto the aneurismal wall can be observed near the distal neck. The flow then leaves the aneurysm sac via the proximal neck. After stent deployment, blood enters the aneurysm near the proximal neck through the pores and leaves the sac near the distal neck. The flow speed near the apex is reduced after stenting. The 3D vector diagrams clearly indicate the change in flow patterns near the neck due to the deployment of the flow-diverting stent (Fig. 4b), which compare favorably with the experimental results in the literature [23].

**Fig. 5:**
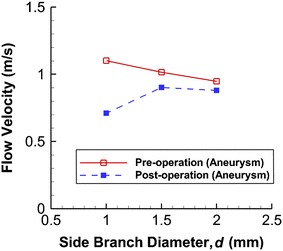
Changes in flow velocity within aneurysm as function of side branch diameter. Peak velocity fails to drop significantly after treatment when side branch diameter is large (*d* = 2.0 mm)

Figure 5 depicts the maximum flow velocity inside the aneurysm for various side branch diameters. Generally speaking, the flow velocity decreased after stent deployment. For *d* = 1.0 mm, the velocity drops from 1.102 m s^−1^ (before stent deployment) to 0.711 m s^−1^ (after stent deployment), a 35.5 % reduction. For a larger side branch diameter (*d* = 2.0 mm), the change from 0.948 to 0.880 m s^−1^ is only 7.2 %. The efficacy of the stent may thus be lower.

This phenomenon can be explained in terms of fluid mechanics. In general, the flow resistance in the side branch increases with decreasing side branch diameter. A higher volume of fluid flux is then diverted to the distal parent artery. However, the presence of a stent at the neck has a large obstruction effect if the flow into the aneurysm is strong. A more dramatic flow speed reduction is thus expected for a smaller side branch diameter.

For the *d* = 2.0 mm case, the maximum pressure (94.4 mmHg) inside the aneurysm decreased by 2.3 mmHg after stenting. For *d* = 1.0 mm, this maximum pressure (98.4 mmHg) dropped by 4.5 mmHg after stent deployment.

For the pressure drop between the inlet (“A”, Fig. 1a) and the outlets (“B” and “C”, Fig. 1a), the drop between “A” and “B” is the same as that between “A” and “C”, since the same pressure condition was assumed at the two outlets. For *d* = 2.0 mm, the pressure drop before stenting (11.5 mmHg) increased by 0.5 mmHg after stent deployment (4.3 % increase). For *d* = 1.0 mm, the pressure drop (15.1 mmHg) decreased by 0.5 mmHg after stenting (3.3 % reduction). The stent might change the overall resistance of blood flow in this bifurcation model. Reduction of normal blood supply may lead to tissue ischemia and occlusion of the side branch vessel has been observed clinically [7].

#### Wall Shear Stress

**Fig. 6:**
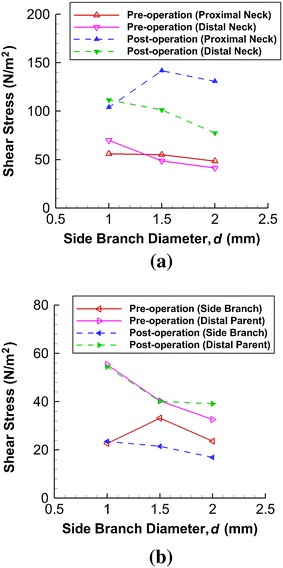
Maximum WSS at various locations varies with side branch diameter. **a** At distal neck, shear stress decreases with increasing side branch diameter under both pre-stenting and post-stenting conditions. **b** When *d* is large, such stress at the side branch vessel shows great drop after stenting, creating undesirable hemodynamic environment

Wall shear stress (WSS) is important in vascular biology and endothelial cell behavior. A low WSS may be undesirable and is related to atherosclerosis and plaque formation [29]. The peak WSS is analyzed for the present model to study the rupture risk of aneurysms. The peak WSS values are found to be located near the neck of the aneurysm, since the stent is situated adjacent to the neck. The variation of WSS at both the proximal and distal regions of the neck for various side branch diameters is depicted in Fig. 6a. Before surgical intervention, the WSS is lower when the side branch diameter is larger, and may be associated with a higher risk clinically [30]. After stent deployment, the WSS values at both the proximal and distal necks increase. However, for *d* = 2.0 mm, the WSS at the distal neck remains below the threshold of safety [29, 30], and constitutes an unfavorable environment in terms of hemodynamics.

Regarding the WSS on the distal parent artery and the side branch vessel (Fig. 6b), the values remain almost unchanged after stenting for *d* = 1.0 mm. However, for *d* = 2.0 mm, the WSS on the side branch vessel decreases from 23.6 to 16.8 N m^−2^ (28.8 % reduction), another indicator of the formation of an unfavorable environment in terms of hemodynamics.

#### Stent Migration Force Per Unit Stent Area

The risk of stent migration is of clinical interest, as it might lead to treatment failure since the aneurysm neck is no longer covered [31]. To investigate this risk, the tangential force (per unit stent area) acting on the stent along the flow direction was determined. For stent area, only the metallic portion is taken into account and the porous portions (or holes) are excluded. The tangential force reported here is the spatial average over the whole stent surface. The force occurs at the instant of the peak systole. The migration force per unit stent area increases almost linearly from 39.7 to 47.3 N m^−2^ (19.1 % increment) when the side branch diameter changes from 1.0 to 2.0 mm, another factor against the *d* = 2.0 mm case.

#### Mean Volume Flow Rates

##### Flow into Aneurysm

**Fig. 7:**
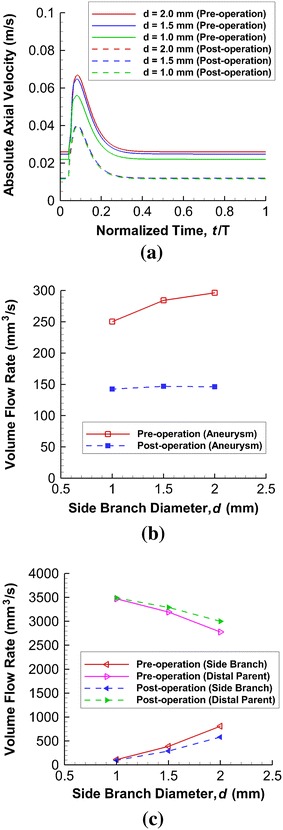
**a** Absolute axial velocity of blood flow at orifice throughout complete cardiac cycle (normalized with respect to cardiac period T). Velocity is generally higher when side branch diameter is large. **b** CFD-derived mean volume flow rate into intracranial aneurysm as function of side branch diameter. A large side branch diameter (d = 2.0 mm) shows a greater flow rate into aneurysm before stent deployment. **c** Corresponding results for side branch vessel and distal parent artery; at small side branch diameter, alteration of mean volume flow rate due to stenting, however, is minimal

Ideally, a flow-diverting stent should hinder the volume flux into the aneurysm with minimal disruption to the blood supply in the side branch vessel. The flow rate into the aneurysm can be measured by creating a control interior surface at the neck connecting the aneurysm and the distal parent vessel. As the aneurysm is a blind sac, the mass of fluid flowing in must be equal to that flowing out. The mean volume flow rate into the aneurysm (*Q*) can be determined by evaluating the following integral [20]:

4 $$ Q = \frac{1}{T}\int_{T}^{t + T} {\frac{\left| V \right|A}{2}dt} = \frac{A}{2T}\int_{T}^{t + T} {\left| V \right|dt} $$

where *T* is the cardiac cycle, |*V*| is the spatial-averaged absolute axial velocity (the component perpendicular to the control surface) at the neck region, *A* is the cross-sectional area of the neck, and *t* is an arbitrary time instant. Simpson’s rule is employed. The absolute axial velocity |*V*| is substantially reduced after stenting (Fig. 7a).

Before stenting, a counterintuitive feature is that for a smaller side branch diameter, there is a smaller flow rate into the aneurysm (Fig. 7b). A smaller side branch has a larger resistance and thus more flow should actually go to the larger distal parent artery. A larger flow into the aneurysmal sac is thus expected, and thus an explanation must be given as to why a reduction is observed instead. A higher velocity in the distal parent vessel produces a fluid jet which hits an attachment point in the aneurysmal sac near the distal neck (Fig. 4a), resulting in less flow into the sac. On the other hand, a slightly smaller velocity in the parent artery allows fluid to enter the sac before it reaches the distal wall. This scenario gives rise to a jet that penetrates further into the sac with a higher attachment point or more influx. A more elaborate explanation would need consideration of secondary streamlines and the distribution patterns of the axial velocity vector in a curved tube.

With the deployment of the stent, the flow rate into the aneurysm drops for all side branch diameters. The stent is effective in impeding the blood flow into the aneurysm, and the chance of thrombosis is enhanced.

##### Flow in Side Branch

Blood flow is directed to the distal parent artery after the implantation of the stent and the flow rate in the side branch vessel is reduced (Fig. 7c). The reductions in the mean volume flow rates in the side branch vessel after stent deployment are 15.5 mm^3^ s^−1^ (0.4 %) for *d* = 1.0 mm, and 226.9 mm^3^ s^−1^ (6.3 %) for *d* = 2.0 mm, out of a total influx into this Y-shaped model of 3586.4 mm^3^ s^−1^.

The streamline configurations, WSS, and volume flow rates constitute a complex dynamic system of fluid flows. The flow in the distal parent artery is probably not a fully developed laminar flow, as the fluid has just flowed through a curved tube at the arterial bifurcation. Secondary streamlines and the highly pulsatile nature of the flow generate complex patterns for the shear stress, which cannot be interpreted in terms of steady flow along a straight pipe. Moreover, the flow in the distal parent vessel might be distorted due to the presence of the aneurysm.

##### Stent Porosity

To investigate the effect of stent porosity on the volume flow rate, the 2-stent case for the idealized models with various side branch diameters is considered. The 2-stent case is modeled by reducing the porosity of the stent to about 35 %, mimicking the deployment of two pipeline stents at the same location clinically [6, 7]. The volume flow rates into the aneurysm and the daughter vessels are summarized in Table 3. After deployment, the flow rate into the aneurysm becomes almost constant for all side branch diameters. This phenomenon is consistent with that in the 1-stent case, but the magnitudes are dramatically smaller in the 2-stent case as the porosity is now much lower.

**Table 3:** Volume flow rates into aneurysm and in daughter vessels for idealized models under post-operation conditions with 2-stent situation

Side branch diameter, *d* (mm)	Stent deployment	Mean volume flow rate (mm^3^ s^−1^)		
		Aneurysm	Side branch vessel	Distal parent artery
1.0	Post-operation with 2-stent situation	41.3	45.8	3540.6
1.5	Post-operation with 2-stent situation	42.1	142.4	3444.0
2.0	Post-operation with 2-stent situation	40.0	221.7	3364.7

The flow rates in the side branch vessel decreased dramatically and slightly for the *d* = 2.0 and 1.0 mm cases, respectively. The flow rate reductions compared with the pre-stenting situation are 1.9 % (*d* = 1.0 mm) and 16.4 % (*d* = 2.0 mm) of the total influx. While the 2-stent situation might be beneficial to the flow reduction inside the aneurysm, this low-porosity configuration may lead to further reduction in the blood supply in the side branch vessel. Again, the *d* = 2.0 mm case seems to create a more unfavorable environment.

### Study of Patient-Specific Aneurysm Models

**Fig. 8:**
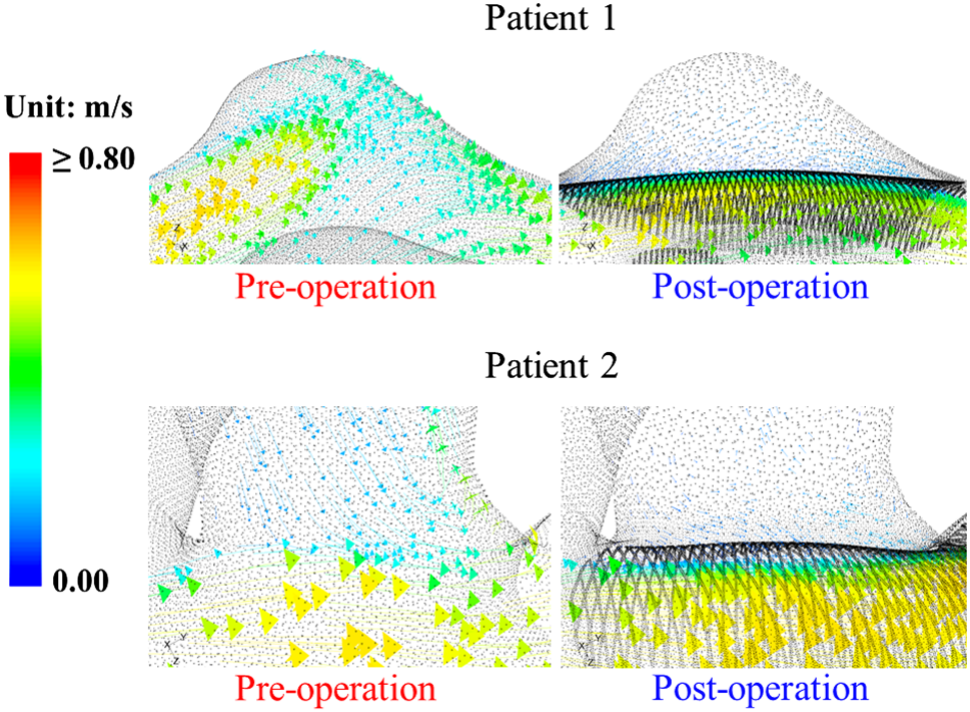
3D vector diagrams near aneurysm neck for two patient-specific models. Drastic flow reduction inside aneurysm is observed in both patients after stent deployment

Two patient-specific bifurcation aneurysms with linear dimensions similar to that of the idealized model, i.e., side branch diameter of about *d* = 1.0 mm, are investigated in this study. The 3D velocity vector plots of both patients before and after stent placement are similar to those observed for the idealized model (Fig. 8). In addition, the flow-diverting stent can dramatically reduce the volume influx into the aneurysm. The maximum pressure inside the aneurysm also drops after stenting for both patients, with reductions of 0.4 and 0.3 mmHg for Patient 1 and Patient 2, respectively. The trend and magnitude of these data are consistent with the conclusions made in Sect. 3.1.1.

**Fig. 9:**
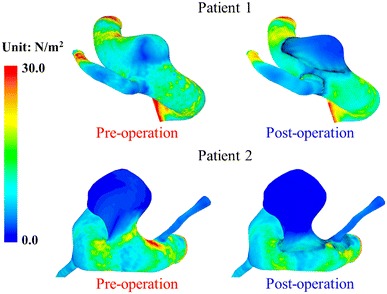
WSS contour plots for aneurysmal and the vessel walls for both patients before and after stent implantation

The trend and magnitude of the changes in WSS patterns on the aneurysmal wall and the vessel walls before and after stent treatment for both patients are similar to those in the idealized model (Fig. 9). At the distal neck of the aneurysm, the shear stress increases slightly for both patients, with peak WSSs going from 20.9 to 22.3 N m^−2^ for Patient 1 and from 19.9 to 20.6 N m^−2^ for Patient 2. The WSS in the side branch vessel is only slightly altered due to stenting, changing from 25.4 to 25.0 N m^−2^ for Patient 1 and from 4.0 to 3.9 N m^−2^ for Patient 2. The WSS in the side branch of Patient 2 is much smaller, since the other side branch (“B*” in Fig. 2) drains a portion of the influx away. This provides further evidence that the WSS on a small side branch would remain almost unchanged after stenting.

The risk of stent migration characterized by the tangential force per unit stent area was determined for both patients. The forces per unit area are 12.1 N m^−2^ (for Patient 1) and 16.8 N m^−2^ (for Patient 2). This tangential force is highly dependent on the neck size and the curvature of the distal parent artery near the neck, and thus direct comparison with the idealized models might not be suitable. However, these values still support the claim that a smaller side branch diameter might have a smaller stent migration force, which is more favorable for stent treatment.

The mean volume flow rates into the aneurysm and in the downstream branches for the patient-specific models are summarized in Table 4. As Patient 1 possesses a wide-necked aneurysm, the inflow rate into the aneurysm is much larger than that of Patient 2. The flow reduction in a saccular aneurysm (Patient 2) is compared with the idealized models. After endovascular treatment, the flow rate into the aneurysm is reduced by about 49 % (from 456.1 to 232.2 mm^3^ s^−1^), which is comparable with the idealized models. For *d* = 1.0 mm, the flow rate into the aneurysm is reduced by 43 % (from 250.4 to 142.5 mm^3^ s^−1^). Consequently, flow rate reductions are similar for both idealized and patient-specific models, despite a quite substantial difference in aneurysm morphology.

**Table 4:** Volume flow rates into aneurysm and in daughter vessels for two patient-specific models with pre-operation and post-operation conditions

Patient model	Stent deployment	Mean volume flow rate (mm^3^ s^−1^)			
		Aneurysm	Side branch vessel 1 “B”	Side branch vessel 2 “B*”	Distal parent artery “C”
Patient 1	Pre-operation	1585.8	212.0	–	3374.4
	Post-operation	285.8	184.4	–	3402.0
Patient 2	Pre-operation	456.1	18.4	94.0	3474.0
	Post-operation	232.2	14.7	92.2	3479.5

Locations of A, B, B*, and C are defined in Fig. 2

The flow rate reductions in the side branch vessel due to stenting in both patient-specific models show quite precise agreement with the idealized models. For the patient-specific models, the flow rates in the side branch vessel (“B” in Fig. 2) reduce slightly from 5.9 to 5.1 % (Patient 1) and from 0.52 to 0.41 % (Patient 2), where the values refer to the percentage of influx. This is consistent with the conclusion drawn in Sect. 3.1.4 that a small side branch diameter would not have drastic flow rate reduction in the side branch vessel due to stenting, preventing damage of the downstream tissues and organs. The good agreement between idealized and patient-specific models is reassuring.

### Study of Peripheral Resistance

The results of the previous sections are based on the assumption that the pressure boundary conditions at the two outlets are identical. This assumption is frequently made in the literature, with many authors prescribing traction-free boundary conditions at multiple outlets [32]. However, the downstream pressures might be distinct in reality due to different peripheral resistance at the outlets. Zhang et al. simulated the downstream resistance by extending the outlet of the model [17]. To study the effect of boundary conditions, the pressure at the outlet of the side branch was reduced relative to that of the distal artery, to simulate the effects of enhanced flow (Table 5). The rate of increase of flow in the side branch as a percentage of the total influx was then studied for various diameters. For a total inflow of 3586.4 mm^3^ s^−1^ in the pre-stenting condition, flow in the side branch can reach 1857.8 mm^3^ s^−1^ (51.8 % of the total) for the *d* = 2.0 mm case if the pressure there is 6 mmHg smaller than that at the other outlet. The corresponding values for *d* = 1.5 and 1.0 mm are 25.0 and 7.6 %, respectively. Moreover, flow in the side branch can only reach 1499.3 mm^3^ s^−1^ (41.8 % of the total) and 458.7 mm^3^ s^−1^ (12.8 % of the total) for *d* = 1.5 and 1.0 mm, respectively, even for an almost unrealistic pressure differential of 15 mmHg between the side branch and the other outlet. These data indicate that the flow rate ratio between two downstream branches is primarily governed by the size of the blood vessel (Table 5).

**Table 5:** Peripheral resistance study of idealized models conducted by varying pressure differential between side branch vessel and distal parent artery with pre-stenting condition

Side branch diameter, *d* (mm)	Pressure boundary conditions		Mean volume flow rate (mm^3^ s^−1^)		
	Side branch vessel	Distal parent artery	Aneurysm	Side branch vessel	Distal parent artery
2.0	P	P	296.3	809.6	2776.8
	P–2 mmHg	P	337.6	1195.4	2391.0
	P–4 mmHg	P	349.0	1541.5	2044.9
	P–6 mmHg	P	339.3	1857.8	1728.6
1.5	P	P	284.2	389.7	3196.7
	P–6 mmHg	P	370.0	896.9	2689.5
	P–15 mmHg	P	379.6	1499.3	2087.1
1.0	P	P	250.4	112.7	3473.7
	P–6 mmHg	P	320.6	271.0	3315.4
	P–15 mmHg	P	378.0	458.7	3127.7

Volume flow rates into aneurysm and in two downstream branches for various side branch diameters are reported (“P” stands for pressure waveform shown in Fig. 3)

In addition, the flow rate into the aneurysm was investigated. When both the side branch vessel and the distal parent artery are under the same pressure conditions, the flow rates into the aneurysm are 296.3 mm^3^ s^−1^ (*d* = 2.0 mm, 8.3 % of the total inflow), 284.2 mm^3^ s^−1^ (*d* = 1.5 mm, 7.9 %), and 250.4 mm^3^ s^−1^ (*d* = 1.0 mm, 7.0 %). When the pressure of the side branch vessel is 6 mmHg smaller than that at the distal parent artery, the flow rates into the aneurysm are 339.3 mm^3^ s^−1^ (*d* = 2.0 mm), 370.0 mm^3^ s^−1^ (*d* = 1.5 mm), and 320.6 mm^3^ s^−1^ (*d* = 1.0 mm). The difference is not large, but the smaller vessel (*d* = 1.0 mm) may have a better clinical outcome, if one associates a slower aneurysmal flow with a higher chance of thrombus formation.

For patient-specific models with dimensions similar to the idealized ones, the pressure boundary conditions were varied to test whether similar trends hold (Table 6). The pressure of side branch vessel 1 (“B”, Fig. 2) of both patient models was set to be 2 mmHg smaller than that of the distal parent artery (“C”, Fig. 2). The flow rates into the aneurysm under these pressure conditions are 1570.3 mm^3^ s^−1^ (Patient 1) and 465.9 mm^3^ s^−1^ (Patient 2), compared to the original flow rates of 1585.8 mm^3^ s^−1^ (Patient 1) and 456.1 mm^3^ s^−1^ (Patient 2) when the pressure at the side branch vessel is the same as that at the distal parent artery. Hence, the flow rates into the aneurysm are only changed slightly when the side branch pressure is varied, consistent with the trend found above.

**Table 6:** Peripheral resistance study of patient-specific models conducted by varying pressure differential between side branch vessel 1 and distal parent artery with pre-stenting condition

Patient model	Stenting deployment	Pressure boundary conditions			Mean volume flow rate (mm^3^ s^−1^)			
		SBV1 “B”	SBV2 “B*”	DPA “C”	An	SBV1 “B”	SBV2 “B*”	DPA “C”
Patient 1	Pre-operation	P	–	P	1585.8	212.0	–	3374.4
	Pre-operation	P–2 mmHg	–	P	1570.3	341.4	–	3245.0
Patient 2	Pre-operation	P	P	P	456.1	18.4	94.0	3474.0
	Pre-operation	P–2 mmHg	P	P	465.9	102.2	73.0	3411.2

Volume flow rates into aneurysm and in downstream branches are reported. Locations of A, B, B*, and C are defined in Fig. 2 (“P” stands for pressure waveform shown in Fig. 3)

*An* aneurysm, *SBV1* side branch vessel 1, *SBV2* side branch vessel 2, *DPA* distal parent artery

Regarding volume flux, the flow rates in the side branch vessel are 341.4 mm^3^ s^−1^ (Patient 1, 9.5 % of a total inflow of 3586.4 mm^3^ s^−1^) and 102.2 mm^3^ s^−1^ (Patient 2, 2.8 %). For identical pressure at the outlets, the corresponding data are 212.0 mm^3^ s^−1^ (5.9 %) and 18.4 mm^3^ s^−1^ (0.5 %). Consequently, with a decrease in side branch pressure, a major portion of blood (more than 90 % of influx) flows into the distal parent artery. These data show very good agreement with the idealized model as the dimensions of the patient-specific models are around *d* = 1.0 mm as well.

### Limitations and Improvements

The merits of CFD analysis include a relatively low cost, noninvasiveness, and information on hemodynamic quantities that are difficult to measure otherwise, e.g., WSS and pressure [33–36]. Difficulties and limitations of most conventional CFD simulations are resolution requirements in modeling complex, 3D, pulsatile flows [37]. Non-Newtonian and other realistic fluid physics effects of blood are also neglected. From the perspective of physiology, blood flow autoregulation in the vessel due to flow rate reduction and the correlation between flow rate reduction and tissue ischemia still require further investigations both clinically and using CFD.

For future works, further computational studies that employ more patient-specific models can provide more clinical insight into the hemodynamics in real patient anatomy. In addition, these computational results ought to be verified by performing in vitro experiments, with phantom models fabricated using rapid prototyping techniques. Flow visualization via ultrasound imaging can increase our understanding of flow dynamics under this pathological condition. Fluid–structure interaction analysis should also be conducted in the future to predict aneurysmal flow more accurately.

## Conclusion

Idealized Y-shaped models with various side branch diameters were utilized to study the hemodynamics of aneurysms near arterial bifurcations, both before and after flow diverter deployment. The likelihood of creating an undesirable hemodynamic environment is correlated with the relative size of the side branch and parent artery. Patient-specific configurations of similar dimensions were investigated. The results exhibit good agreement with those from idealized models. In post-stenting configurations, lower WSS was observed at the distal neck of the aneurysm when the side branch diameter increased. Small WSS has frequently been associated with enhanced risk of aneurysm growth and rupture [29].

Furthermore, the flow rate into the aneurysm before stenting increases when the side branch diameter increases. Enhanced flow rate in the aneurysmal sac is usually associated with lower chance of thrombosis [23]. As the side branch diameter increases, the percentage drop in flow rate in that branch after flow diverter deployment becomes larger. Eventually, occlusion may result in that vessel. This scenario has been observed in many clinical studies, e.g., cases involving aneurysm in the vicinity of the ophthalmic artery [7].

In conclusion, the present work can assist clinical personnel in assessing the hemodynamics for a range of side branch diameters for aneurysms located near bifurcations. The post-treatment condition might also be predicted by virtual stenting prior to intervention. This computational analysis may be used to identify patients who might not respond well to PED deployment mechanisms in advance. Hence, the risk of extensive and expensive remedial procedures may be mitigated.
